# 

**Published:** 1905-01-21

**Authors:** 


### NOTICE TO CORRESPONDENTS.

All MSS., Letters, Books for Review, and other matters intended for the Editor should be addressed THE EDITOR, "THE HOSPITAL." THE HOSPITAL BUILDINGS, 28 & 29 SOUTHAMPTON STREET, STRAND, W.C.

The Editor cannot undertake to return rejected MS., even when accompanied by stamped directed envelope

**Notice to Advertisers and Subscribers.**

All advertisements, Orders for Copies of THE HOSPITAL, and Business Communications should be addressed to **THE MANAGER (Not to the Editor),** THE HOSPITAL, 28 & 29 Southampton Street, Strand, London, W.C.

The Cost of Subscription, post free, is as follows :—Per week, 3½d.; per quarter, 3s. 3d.; per half-year, 6s. 6d.; per annum, 13s. Foreign and Colonial —Per annum, 19s. 6d., payable, in all cases, in advance.

**NOTICE.—Vol. XXXVI. of "The Hospital" (April to September 1904), in handsome cloth binding, is now on sale at the office of this Journal, price 10s. 6d. Cases for binding the Numbers contained in this Volume 2s. Index to Vol. XXXVI., 3½d., post free.**

**The Monthly part for January is now ready. Price 1s., by post, 1s. 3d.**

### BOOKS FOR

### Midwives & Maternity Nurses.

*Published by* **THE SCIENTIFIC PRESS, LTD.**

| | |
|---|---|
| **A COMPLETE HANDBOOK OF MIDWIFERY.**<br>By J. K. WATSON, M.D.<br>Designed to meet the requirements of the Central Midwives Board.<br>Containing over 350 pp., and Illustrated with 143 Diagrams. Cloth. | 6/- net,<br>6/4 post free. |
| **A PRACTICAL HANDBOOK OF MIDWIFERY.**<br>By FRANCIS W. HAULTAIN, M.D.<br>A valuable work for Practising Midwives.<br>New Edition. Illustrated. Bound in leather. | 6/- net,<br>6/3 post free. |
| **NURSING NOTES ON MIDWIFERY AND GYNÆCOLOGY.**<br>By SISTER ROSS, Rotunda Hospital.<br>Suitable size for the apron pocket. Cloth. | 2/- net,<br>2/1 post free. |
| **THE MIDWIVES' POCKET-BOOK.**<br>By HONNOR MORTEN. Cloth. | 1/- post free. |
| **HOW TO BECOME A CERTIFIED MIDWIFE.**<br>By E. L. C. APPEL, M.B. Limp cloth. | 1/- net,<br>1/1 post free. |
| **PRELIMINARY LECTURES ON MIDWIFERY.**<br>By MARGARET M. CALEY, late Matron Fulham Midwifery School.<br>Cloth. | 1/6 net,<br>1/8 post free. |
| **DISTRICT MIDWIFERY CASE-BOOK.**<br>Compiled by MARGARET M. CALEY. Suitable size for the pocket. | 6d. net,<br>7d. post free. |

***N.B.—All Forms and Books needed in accordance with the requirements of the new Midwives Act also Supplied.***

**The Scientific Press, Ltd.,** *Publishers and Booksellers of all Classes of Hospital Nursing Publications, Charts, Case Books, &c.*

**28 & 29 Southampton Street, Strand, London, W.C.**

**Complete Catalogue of Nursing Manuals post free on application.**

